# Prevalence of maternal antenatal anxiety and its association with demographic and socioeconomic factors: A multicentre study in Italy

**DOI:** 10.1192/j.eurpsy.2020.82

**Published:** 2020-09-07

**Authors:** L. Cena, F. Mirabella, G. Palumbo, A. Gigantesco, A. Trainini, A. Stefana

**Affiliations:** 1 Department of Clinical and Experimental Sciences, Section of Neuroscience, Observatory of Perinatal Clinical Psychology, University of Brescia, viale Europa 11, Brescia 25123, Italy; 2 Center for Behavioural Sciences and Mental Health, National Institute of Health, Viale Regina Elena 299, Rome 00161, Italy

**Keywords:** Demographic factors, maternal antenatal anxiety, screening, socioeconomic factors

## Abstract

**Background.:**

Maternal antenatal anxiety is very common, and despite its short- and long-term effects on both mothers and fetus outcomes, it has received less attention than it deserves in scientific research and clinical practice. Therefore, we aimed to estimate the prevalence of state anxiety in the antenatal period, and to analyze its association with demographic and socioeconomic factors.

**Methods.:**

A total of 1142 pregnant women from nine Italian healthcare centers were assessed through the state scale of the State–Trait Anxiety Inventory and a clinical interview. Demographic and socioeconomic factors were also measured.

**Results.:**

The prevalence of anxiety was 24.3% among pregnant women. There was a significantly higher risk of anxiety in pregnant women with low level of education (*p* < 0.01), who are jobless (*p* < 0.01), and who have economic problems (*p* < 0.01). Furthermore, pregnant women experience higher level of anxiety when they have not planned the pregnancy (*p* < 0.01), have a history of abortion (*p* < 0.05), and have children living at the time of the current pregnancy (*p* < 0.05).

**Conclusion.:**

There exists a significant association between maternal antenatal anxiety and economic conditions. Early evaluation of socioeconomic status of pregnant women and their families in order to identify disadvantaged situations might reduce the prevalence of antenatal anxiety and its direct and indirect costs.

## Introduction

Maternal antenatal anxiety and related disorders are very common [1,2], and despite it being frequently comorbid with [3,4], and possibly more common than, depression [1,5], it has received less attention than it deserves in scientific research and clinical practice. Moreover, parental prenatal complications can interfere with the parent–child relationship, with the risk of significant consequences over the years for the child’s development [6,7]. From a clinical point of view, this is a considerable omission given the growing evidence that antenatal maternal anxiety can cause adverse short-term and long-term effects on both mothers and fetal/infant outcomes [8–16], including an increased risk for suicide and for neonatal morbidity, which are associated with significant economic healthcare costs [17]. The prevalence of anxiety during pregnancy is high worldwide (up to approximately 37%); however, in low- and middle-income countries, it is higher than in high-income countries [1,2], with heterogeneity across nations with comparable economic status.

Several studies have investigated the relationship between demographic and socioeconomic risk factors with antenatal anxiety [2,18]. The results showed that several demographic (e.g., maternal age) and socioeconomic factors (e.g., employment, financial status) were associated with differences in the prevalence of anxiety symptoms or disorders, but the results are equivocal. However, both the prevalence and the distribution of these protective and risk factors may change over time, especially in a period of major socioeconomic change [19,20], such as the global economic crisis beginning in 2008, which led to the increased consumption of anxiolytic drugs and antidepressants with anxiolytic properties [21], to a decline in the number of births [22] and to impaired development in medical, scientific, and health innovations [23] that, in the next few years, could reduce the availability of help for families and health services [24]. However, despite the recently available and growing research evidence highlighting the need for early identification [25] and prompt treatment of maternal anxiety during both pregnancy and the postpartum period, anxiety remains largely undetected and untreated in perinatal women in Italy.

The aims of this study were (a) to assess the prevalence of state anxiety in the antenatal period (further stratified by trimesters) in a large sample of women attending healthcare centers in Italy and (b) to analyze its association with demographic and socioeconomic factors.

## Methods

### Outline of the study

The study was conducted as part of the “Screening e intervento precoce nelle sindromi d’ansia e di depressione perinatale. Prevenzione e promozione salute mentale della madre-bambino-padre” (Screening and early intervention for perinatal anxiety and depressive disorders: Prevention and promotion of mothers’, children’s, and fathers’ mental health) project [26] coordinated by the University of Brescia’s Observatory of Perinatal Clinical Psychology and the Italian National Institute of Health (Istituto Superiore di Sanità, ISS). The main objectives of this Italian multicenter project were to apply a perinatal depression and anxiety screening procedure that could be developed in different structures, as it requires the collaboration and connection between structurally and functionally existing resources, and to evaluate the effectiveness of the psychological intervention of Milgrom and colleagues [27–29] for both antenatal and postnatal depression and/or anxiety in Italian setting. The research project was assessed and approved by the ethics committee of the Healthcare Centre of Bologna (registration number 77808, dated 6/27/2017).

### Study design and sample

We performed a prospective study involving nine healthcare centers (facilities associated with the Observatory of Perinatal Clinical Psychology, University of Brescia, Italy) located throughout Italy during the period, March 2017 to June 2018. The Observatory of Perinatal Clinical Psychology (https://www.unibs.it/node/12195) coordinated and managed the implementation of the study in each healthcare center. Only cross-sectional measures were included in the current analyses because screening for anxiety was carried out at baseline. The inclusion criteria were as follows: being ≥18 years old; being pregnant or having a biological baby aged ≤52 weeks; and being able to speak and read Italian. The exclusion criteria for baseline assessment were as follows: having psychotic symptoms, and/or having issues with drug or substance abuse.

### Data collection

Each woman was interviewed in a private setting by a female licensed psychologist. All psychologists were trained in the postgraduate course of perinatal clinical psychology (University of Brescia, Italy) and were associated with the healthcare center. All the psychologists also completed a propaedeutic training course for the study, developed by the National Institutes of Health, on screening and assessment instruments and on psychological intervention [30]. The clinical interview was adopted to elicit information regarding maternal experience with symptoms of stress, anxiety, and depression. All women completed the interview and completed self-report questionnaires.

### Instruments

#### Psychosocial and Clinical Assessment Form

The Psychosocial and Clinical Assessment Form [31,32] was used to obtain information on demographic and socioeconomic characteristics. In this study, the following demographic variables were considered: age, marital status, number of previous pregnancies, number of abortions, number of previous children (living), planning of the current pregnancy, and use of assisted reproductive technology. The socioeconomic variables were educational level, working status, and economic status.

### State–Trait Anxiety Inventory

Given that the assessment of mental diseases, including antenatal diseases, is based primarily on self-perceived symptoms, evaluating these data using valid, reliable, and feasible self-rating scales can be useful. The state scale of the State–Trait Anxiety Inventory [33–35] was used to evaluate anxiety. It is a self-report questionnaire composed of 20 items that measure state anxiety, that is, anxiety in the current situation or time period. The possible responses to each item are on a 4-point Likert scale. The total score ranges from 20 to 80, with higher scores indicating more severe anxiety. This instrument is the most widely used tool in research on anxiety in women in the antenatal period [1,36]. The construct and content validity of the STAI for pregnant women has been proven [37,38].

### Procedures

Women who met the inclusion criteria were approached by one of the professionals affiliated with the healthcare center and involved in the research when they attended a routine antenatal appointment. They received information about the content and implications of the study. Future mothers who signed the informed consent document completed the questionnaires and then underwent an interview with a clinical psychologist.

### Statistical analysis

All variables were categorized. A statistical analysis that included descriptive and multiple logistic regression models was performed. For descriptive analyses, frequencies and percentages were calculated for categorical variables, and the Chi-square test was utilized for comparisons. The logistic regression model was used to evaluate the associations between the demographic and socioeconomic variables and the risk of antenatal anxiety. In the analytic models, each demographic and socioeconomic variable was included both individually and together. All analyses were performed using the Statistical Package for Social Science (SPSS) version 25.

## Results

### Subjects

To estimate the minimum sample size, we relied on three studies [39–41], indicating that it was necessary to enroll 296 patients. However, our main aim was to recruit as large a sample as possible to promote perinatal mental health; thus, at the end of the 1-year recruitment period, we enrolled more mothers. Among the 2096 women invited to join the study, 619 (29.5%) refused, mainly due to lack of time, personal disinterest in the topic, and the conviction that they are not and never will become anxious or depressed. Therefore, the total study sample consisted of 1,477 women. Of these, 28 women did not complete the anxiety questionnaire. Thus, the sample includes 1,142 pregnant women and 307 new mothers. Given the aims of this study, only pregnant women were included in the current statistical analysis. Table 1 presents the list of the healthcare centers in which the pregnant women were recruited. Table 2 presents demographic and socioeconomic characteristics, along with an estimation of the relative risk of anxiety through both bivariate and multivariate analyses.

**Table 1.** Healthcare centers involved in the study (facilities associated with the Observatory of Perinatal Clinical Psychology, University of Brescia, Italy).

Location	Name	Unit type	Professionals involved
Treviolo (Bergamo)	Mani di Scorta	Clinic and Family Center	1 PsyD 1 Midwife
Bologna	Maggiore Hospital	Normal Pregnancy and Breastfeeding Department	1 PsyD 1 Gynecologist 2 Midwives
Brescia	Clinical Institute City of Brescia	Obstetrics and Gynecology OU	2 PsyD 1 PT in training
Enna	Umberto I Hospital	Obstetrics and Gynecology OUC, Normal Pregnancy Clinic	1 PsyD
Florence	LHA of Toscana Centro	Family Clinic and Pediatric Surgery	4 PsyD
Mantova	Carlo Poma Hospital	Clinical Psychology Department; NICU	1 PsyD 1 Midwife 1 Nurse
Milan	San Giuseppe Hospital	Obstetrics and Gynecology OU	1 PsyD
Novara	GruppoPsychè Association, Maggiore della Carità Hospital	Obstetrics and Gynecology OU	3 PsyD
Rome	Cristo Re Hospital	Obstetrics and Gynecology OUC	5 PsyD 1 Psychologist

Abbreviations: CN, Child Neuropsychiatrist; LHA, Local Health Authority; NICU, Neonatal Intensive Care Unit; OU, Unit/Department; OUC, Operating Unit Complex; PsyD, Psychologist–Psychotherapist; RHS, Regional Health Service.

**Table 2.** Demographic and socioeconomic characteristics of the sample, prevalence of anxiety risk (STAI), and multiple logistic regression model.

		Sample *n* (%)	STAI ≥40 *n* (%)	Exp(B) (IC95%)	*p* value	Exp(B)a (IC95%)	*p* value
Age	18–29	269 (23.6)	79 (29.4)	Ref.		Ref.	
	30–35	536 (47.0)	116 (21.6)	0.66 (0.48–0.93)	0.02	0.98 (0.67–1.43)	0.91
	>35	335 (29.4)	81 (24.2)	0.77 (0.53–1.10)	0.15	0.93 (0.70–1.63)	0.76
Nationality	Italian	1063 (93.1)	262 (24.6)	Ref.		Ref.	
	Non-Italian	79 (6.9)	15 (19.0)	0.72 (0.40–1.28)	0.26	0.59 (0.31–1.13)	0.11
Marital status	Married or cohabiting	1039 (91.6)	246 (23.7)	Ref.		Ref.	
	Single, separated, divorced, or widowed	95 (8.4)	30 (31.6)	0.67 (0.43–1.06)	0.09	0.86 (0.53–1.40)	0.55
Educational level	University	581 (51.3)	120 (20.7)	0.34 (0.23–0.50)	<0.01	0.60 (0.37–0.96)	0.03
	Secondary	411 (36.3)	94 (22.9)	0.39 (0.26–0.58)	<0.01	0.57 (0.36–0.89)	0.01
	Primary or Illiterate	141 (12.4)	61 (43.3)**	Ref.		Ref.	
Working status	Permanent employee	814 (72.1)	176 (21.6)	0.44 (0.31–0.61)	<0.01	0.64 (0.43–0.95)	0.02
	Temporary employee	114 (10.1)	21 (18.4)	0.36 (0.20–0.62)	<0.01	0.47 (0.26–0.84)	0.01
	Student, homemaker or unemployed	201 (17.8)	78 (38.8)**	Ref.		Ref.	
Economic status	Average high status	519 (46.1)	124 (23.9)	0.45 (0.27–0.74)	<0.01	0.58 (0.33–1.00)	0.05
	A few problems without specific difficulties	534 (47.3)	120 (22.5)	0.41 (0.25–0.68)	<0.01	0.52 (0.30–0.88)	0.01
	Same/many problems	75 (6.6)	31 (41.3)**	Ref.		Ref.	
Planned pregnancy	Yes	797 (70.7)	157 (19.7)	Ref.		Ref.	
	No	330 (29.3)	116 (35.2)**	0.45 (0.34–0.60)	<0.01	0.57 (0.42–0.78)	<0.01
Resort to assisted reproductive technology	Yes	83 (7.4)	12 (14.5)	Ref.		Ref.	
	No	1046 (92.6)	263 (25.1)*	0.50 (0.27–0.94)	0.03	0.58 (0.33–1.00)	0.05
Previous pregnancies	Yes	287 (25.1)	79 (27.5)	Ref.		Ref.	
	No	855 (74.9)	198 (23.2)	0.79 (0.93–1.71)	0.14	0.72 (0.43–1.21)	0.22
Past abortion	Yes	296 (26.2)	95 (32.1)*	Ref.		Ref.	
	No	832 (73.8)	180 (21.6)	0.59 (1.27–2.30)	<0.01	0.58 (1.20–2.48)	<0.01
Children living in the time of this pregnancy	Yes	192 (16.8)	59 (30.7)*	Ref.		Ref.	
	No	950 (83.2)	218 (22.9)	0.67 (1.06–2.10)	0.02	0.67 (0.88–2.53)	0.14
Total		1142 (100)	277 (24.3)				

Numbers may not sum to total due to missing data.

Exp(B) = exponentiation of the B coefficient; Exp(B)a = exponentiation of the B coefficient adjusted by all demographic and socioeconomic characteristics variables.

* *p* < 0.05.

** *p* < 0.01.

### Prevalence of antenatal state anxiety

The prevalence of anxiety (Table 3) was 24.3% among pregnant women. A further division into 13-week trimesters was applied, showing that the prevalence of antenatal anxiety was high (36.5%) in the second trimester and then decreased in the third and last trimesters of pregnancy.

**Table 3.** Results of screening for antenatal anxiety risk separated by trimesters and total frequencies and percentages.

Gestational weeks	Women for quarter	STAI-S < 40 *n* (%)	STAI-S ≥ 40 *n* (%)
1–13	2	2 (100.0)	0
14–26	126	80 (63.5)	46 (36.5)
27–40	1,014	783 (77.2)	231 (22.8)
Total sample	1,142 (78.8)	865 (75.7)	277 (24.3)

Bivariate analyses (Table 2) showed a significantly higher risk of anxiety in pregnant women who have a low level of education (primary or semiliterate) (*p*< 0.01), who are jobless (i.e., student, homemaker, or unemployed) (*p*< 0.01), and who have economic problems (*p*< 0.01). Furthermore, during the antenatal period, women experienced a higher level of anxiety when they had not planned the pregnancy (*p*< 0.01), did not resort to assisted reproductive technology (*p*< 0.05), had a history of abortion (*p*< 0.05), and had children living at the time of the current pregnancy (*p*< 0.05).

The adjusted logistic regression analysis (see Table 2) showed that pregnant women with a high (university or secondary) educational level (Exp B = 0.60), temporary or permanent employment (Exp B = 0.64), and, in particular, either a high economic status or few economic problems (Exp B = 0.58) showed a reduction in the risk of antenatal anxiety by almost half. Furthermore, a similar reduction in risk was observed in women who had planned for their pregnancy (Exp B = 0.57).

## Discussion

This study is one of the largest to evaluate the prevalence of anxiety during pregnancy in a sample of women attending healthcare centers in Italy. In general, the fact that the demographic data of participants in this study are comparable to those from population-based epidemiological studies [42] indicates that our results are representative of the overall population of pregnant women in Italy. Our findings are in line with the prevalence in a previous Italian study [43] and the overall pooled prevalence for self-reported anxiety symptoms of 22.9% reported in a recent systematic review and meta-analysis [1]. Similarities in the prevalence of maternal antenatal anxiety remain regardless of which diagnostic tool was used. Regarding the use of the STAI in this study, it should be noted that it is the most widely used self-reporting measure of anxiety. Furthermore, its criterion, discriminant and predictive validity [44], and ease of use can provide a reasonably accurate estimate of prevalence, and its widespread use in research studies [1,16] can enable more accurate comparisons among nations.

With regard to the trimestral prevalence of antenatal anxiety, our study found that the prevalence of anxiety was highest during the second trimester. This observation is inconsistent with the results from a recent meta-analysis [1] that found that the prevalence rate for anxiety symptoms increased progressively from the first to the third trimester as the pregnancy progressed. However, it should be noted that the results regarding the monthly/trimestral/semestral prevalence of perinatal anxiety were not univocal in all studies [1,2].

Our study shows that having a low level of education, being jobless, and having financial difficulties are three crucial predisposing factors of anxiety in pregnant women. These associations are clearly consistent with previous studies that found that antenatal anxiety was more prevalent in women with low education and/or low socioeconomic status (e.g., unemployment, financial adversity) [45–49] and might be related to the global economic crisis that currently affects, especially, southern nations [50]. Studies conducted in developing countries, where low education and low socioeconomic status are both present, highlight the association with prenatal anxiety [51–53].

Furthermore, consistent with previous studies, our results show that antenatal anxiety is more prevalent in women who have unplanned pregnancies [43,54] and who have living children at the time of the current pregnancy [55]. We assume that the reasons for these associations most likely concern the costs associated with raising one or more children, especially when the (new) child is unplanned. This interpretation finds support in the results from previous studies, showing that low income, unemployment, and financial adversity [2] are related to higher levels of antenatal anxiety symptoms. Moreover, it would also explain why resorting to assisted reproductive techniques, which in Italy requires financial resources, was not a risk factor.

Our findings regarding the association between ongoing economic hardships or difficulties and antenatal anxiety can be particularly important in light of the short- and long-term adverse impacts of the coronavirus disease 2019 (COVID-19) pandemic and restrictive measures adopted to counteract its spread [56,57]. Indeed, the COVID-19 outbreak has significantly impacted European and global economies both in the short term and in the coming years [58,59]. Furthermore, as shown by general population surveys, social isolation related to the COVID-19 pandemic is associated with a wide range of adverse psychological effects, including clinical anxiety and depression and concern about financial difficulties [60,61], which can persist for months or years afterward, as indicated by the literature on quarantine [62]. A vulnerable population, such as women in the perinatal period, may be among the individuals who are most affected.

## Clinical Impact

Our findings suggest that screening for early detection of antenatal anxiety (as well as depression, which is frequently comorbid with anxiety [3,4]) is recommended for all pregnant women, but especially for those who have a poor level of education and financial difficulties. Early detection and diagnosis will enable psychological and, where appropriate, pharmacological treatment in the health services to prevent anxiety complications in both these women and their children.

## Limitations

Three main limitations of this study should be noted. First, a cross-sectional approach to antenatal anxiety does not allow us to fully explore whether and what factors may predict persistent anxiety symptoms beginning during pregnancy and progressing to postpartum. Second, the size of the sample during the first trimester of pregnancy was too small to draw any conclusions. Finally, the rates of diagnosis of any anxiety disorder in our sample were not assessed.

## Conclusions

There is a significant association between maternal antenatal anxiety and economic conditions. The aftermath of the great recession of 2008–2009 and the ongoing economic impact of the COVID-19 pose a serious problem for women and their families. With the present historical and economic background in mind, our findings would allow us to hypothesize that early evaluation of the socioeconomic status of pregnant women and their families to identify disadvantaged situations might reduce the prevalence of antenatal anxiety and its direct and indirect costs. In this sense, our findings may give Italian health policy planners useful information to develop new cost-effective antenatal prevention programs focused on socioeconomically disadvantaged families. Furthermore, we believe that our results will serve as a baseline for future comparisons between nations inside and outside the European Union, as well as for new studies on the protective and risk factors related to perinatal anxiety in those nations.

## Data Availability

The complete dataset is available from the corresponding author upon request.
